# Spectral function modulation based on nonlinear frequency division multiplexing

**DOI:** 10.1038/s41598-017-06427-1

**Published:** 2017-07-20

**Authors:** Guangqiang He, Luning Wang, Chenyang Li, Siyu Liu, Weisheng Hu

**Affiliations:** 1 State Key Laboratory of Advanced Optical Communication Systems and Networks, Department of Electronic Engineering, Shanghai Jiao Tong University, Shanghai, 200240 China; 2 State Key Laboratory of Information Security, Institute of Information Engineering, Chinese Academy of Sciences, Beijing, 100093 China

## Abstract

A combination of phase and amplitude modulation in nonlinear discrete spectrum is proposed based on nonlinear frequency division multiplexing. Here the integrable nonlinear Schrodinger equation is used as the channel model. We propose the transmission system with designed transmitting signals and implement our scheme with simulation. We use 8QAM constellation and 2 eigenvalues to generate 5 bit signals, which greatly improve spectral efficiency. This method can be expanded for higher order modulation and further improve transmission capacity in limited bandwidth.

## Introduction

Optical fiber communication is drawing much attention for its prospect in future application with heavy information load. Cloud computing, HD videos and instant news sharing are all prospect applications in 5G communication and are bandwidth hungry, exerting pressure on both channels for spectral efficiency and receiver modules for data accuracy. With the increasing data rates, the capacity of optical fiber systems has been pushed to the theoretical boundary of standard single-mode fiber (SSMF), which is mainly imposed by three factors, namely optical noise, dispersion and nonlinearity. Nonlinear effects pose great threat to widely-used linear transmission technologies, leading to limited capacity and distorted signals. For most linear systems, nonlinearity presents itself as a detrimental effect. So it is of great importance that we find effective methods to removal the nonlinear distortions and support communication with high spectral efficiency.

Extensive efforts have been made to suppress nonlinearity, and some of them prove to work out well. These methods include digital backward propagation (DBP)^1^, optical phase conjugation (OPC)^2, 3^, phase-conjugated twin waves (PCTW)^4, 5^ and coherent optical carriers^6^. However, we still face limitations and challenges in practical application because of flexibility and complexity. For example, DBP requires high time complexity since it is based on the step size along the fiber. Although there are optimized methods^7^ with much larger step size, they increase the complexity of hardware in DSP module. Other methods also show nonnegligible drawbacks.

Thus, original time domain and linear spectral domain are found unsuitable for nonlinearity removal. Recently an alternative method based on nonlinear Fourier transformation (NFT) has showed its huge potential in recovery of nonlinear distortion. Different from other approaches which see nonlinearity as a destructive effect, here fiber nonlinearity is an essential element causing trivial evolution in the spectrum. Similar to linear Fourier transformation (FT) which shows dispersion as a phase rotation in frequency space, NFT casts both the dispersion and nonlinearity as a decoupled item in nonlinear spectrum. Two parts of nonlinear spectrum may be generated through NFT according to the input signal, namely discrete spectrum and continuous spectrum, both of which can be used for modulation and transmission. With careful design of signals, we can generate discrete or continuous spectrum only. Data transmission with discrete spectrum is also referred to as nonlinear frequency division multiplexing (NFDM), and some relative experiments have been conducted^8, 9^. The method based on continuous part of nonlinear spectrum is termed as nonlinear inverse synthesis (NIS)^10^. In recent years both parts have been considered. In this paper we propose a new method of multiplexing using NFDM with the combination of eigenvalues and spectral functions. Here we use two eigenvalues and 8QAM in nonlinear spectrum, producing satisfactory results at the receiver.

This paper is arranged as follows. In theoretical modeling, we derive the mathematical expression of nonlinear Fourier transformation and introduce eigenvalue communication and NFDM. In results, we describe the design of transmitted signals with INFT. The system setup aims to introduce the whole transmission structure. In discussion, we discuss the strengths and weaknesses of the proposed modulation scheme. In method, we introduce the principle of signal design at the transmitter.

## Theoretical Modeling

Eigenvalue communication has been repeatedly proved significant in data communication for nonlinearity removal. It presents stable properties in transmission and provides a rather simple method of nonlinear compensation in a new domain. This concept was first proposed in 1972 by V. E. Zakharov and A. B. Shabat^11^. The process is mainly based on the integrability of nonlinear Schrodinger equation (NLSE) with no loss and no higher-order dispersion. Here we consider the normalized NLSE with constant anomalous dispersion as the master model in our system. Normalized NLSE is given by:

1 $$\frac{\partial q(t,z)}{\partial z}=-j(\frac{{\partial }^{2}q(t,z)}{\partial {t}^{2}}+2q(t,z){|q(t,z)|}^{2})$$

where *q*(*t*, *z*) is the normalized signal along the fiber. It shows that the optical field envelope is varying slowly with propagation. Eigenvalue communication^11–13^ indicates that even though the signal suffers serious distortion in time domain due to dispersion and fiber nonlinearity, its eigenvalues remain almost the same in transmission^14^. Thus signal recovery using eigenvalues is made possible. By using eigenvalues and nonlinear Fourier transformation (Eq. 2),

2 $$\frac{dv}{dt}=(\begin{array}{cc}-j\lambda  & q(t)\\ -{q}^{\ast }(t) & j\lambda \end{array})v,v({T}_{1},\lambda )=(\begin{array}{c}1\\ 0\end{array}){e}^{-j\lambda {T}_{1}}.$$

we can derive the Jost scattering coefficients *a*(*λ*) and *b*(*λ*):

3 $$\begin{array}{rcl}a(\lambda ) & = & {v}_{1}({T}_{2},\lambda ){e}^{j\lambda {T}_{2}}\\ b(\lambda ) & = & {v}_{2}({T}_{2},\lambda ){e}^{-j\lambda {T}_{2}}\end{array}$$

Then we can transform the signal to nonlinear Fourier spectrum (Eq. 4), where nonlinear distortion presents itself as a simple phase rotation^15^.

4 $$q(\lambda )=\{\begin{array}{ll}\frac{b(\lambda )}{a(\lambda )} & \lambda \in {\mathbb{R}}\\ \frac{b(\lambda )}{a^{\prime} (\lambda )} & \lambda \in S\subset {{\mathbb{C}}}^{+}\end{array}$$

Here *λ* and *v* respectively represent eigenvalues and eigenvectors, and *q*(*λ*) is referred to as spectral functions. For continuous spectrum, eigenvalues run through the whole real axis, and its spectral function is *q*(*λ*) = *b*(*λ*)/*a*(*λ*). For discrete spectrum, eigenvalues belong to the isolated zeros of *a*(*λ*) in the upper half of the complex plane. Its spectral function is shown as $q(\lambda )=b(\lambda )/\frac{da(\lambda )}{d\lambda }$. This process is realized by NFT, through which a signal is transformed from time domain to nonlinear spectral domain^16^. In nonlinear spectral domain, the original signal can be easily recovered at the receiver with a phase shift. Inversely if *q*(*λ*) and *λ* are known, we can construct the signal in time domain with inverse nonlinear Fourier transformation (INFT). In this sense NFT and INFT form a transformation pair.

In order to improve the capacity, researchers are extensively investigating nonlinear frequency division multiplexing. There are two degrees of freedom in this approach, namely discrete eigenvalues and spectral functions. Up to now several papers about eigenvalue modulation^17, 18^ have been reported where multiple discrete eigenvalues are used for data transmission. Other groups claim interest in spectral phase modulation^19^. But there have been no reported results about using both phase and amplitude modulation. Since different eigenvalues represent different nonlinear frequency bands, it is predictable that transmission capacity grows linearly with the number of available spectral functions. Here we explicitly introduce the combination of eigenvalue and spectral function modulation as a new transmission scheme.

## Results

### Generation of two-soliton spectral function modulated signals

Both eigenvalue modulation and spectral function modulation are efficient methods of multiplexing by enabling more signals on limited nonlinear bandwidth. Signals with *N* different eigenvalues can be used to generate 0-, 1-, 2-, $\cdots$, N- solitons through various combination. For signals with the same eigenvalue, scaling the amplitude means a shift of waveform center in time domain. So it simplifies the process of signal generation. We only need to generate one set of signals with the same spectral amplitude from INFT and generate the other set with corresponding eigenvalues from shifts in time domain. In our method, different signals are mapped as different bit sequences. For example, *N* waveforms are mapped into *log*_2_ *N* bits.

**Figure 1:**
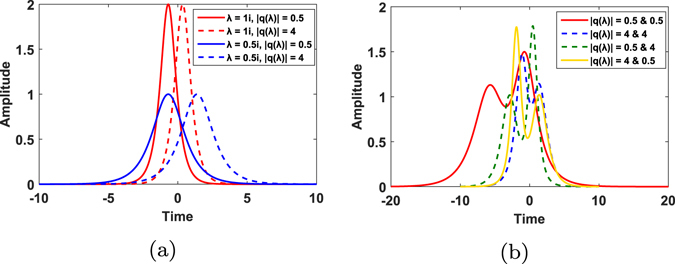
(**a**) Transmitted signals with only one eigenvalue produced by INFT. All the waveforms here are produced with one eigenvalue 1*i* or 0.5*i*, namely one soliton. In the one-soliton case there are totally 16 signals generated by two possible eigenvalues and 8 spectral functions (8QAM). Signals which only differ in spectral phases have the same envelope in time domain. So we have four different envelopes above. (**b**) Transmitted signals with both eigenvalues 1*i* and 0.5*i* produced by INFT. These signals share the same eigenvalues and differ in spectral functions.

We show a combination of two different schemes of modulation, namely eigenvalue modulation and advanced spectral function modulation in nonlinear spectrum domain. Two eigenvalues and 8QAM in nonlinear spectral domain are adopted to generate 32 diverse signals, indicating 5 bits. For signals with the same eigenvalue, scaling the phase also means phase modulation in time domain. The transmitted signals are shown in Fig. 1. Figure 1(a) shows the signals with one eigenvalue (one-soliton) at 1*i* or 0.5*i*. All 16 one-soliton signals totally have 4 diverse envelopes, since signals which differ only in phase have the same envelope in time domain. It also indicates that signals with the same eigenvalue but different spectral amplitudes show only a time shift, which means scaling the amplitude leads to a shift of center in time domain (comparing signals in solid lines and dashed lines with the same color). For two-soliton signals there are 64 possible combinations, from which 16 are chosen for transmission as shown in Fig. 1(b). So these signals differ in corresponding spectral amplitudes.

**Figure 2:**
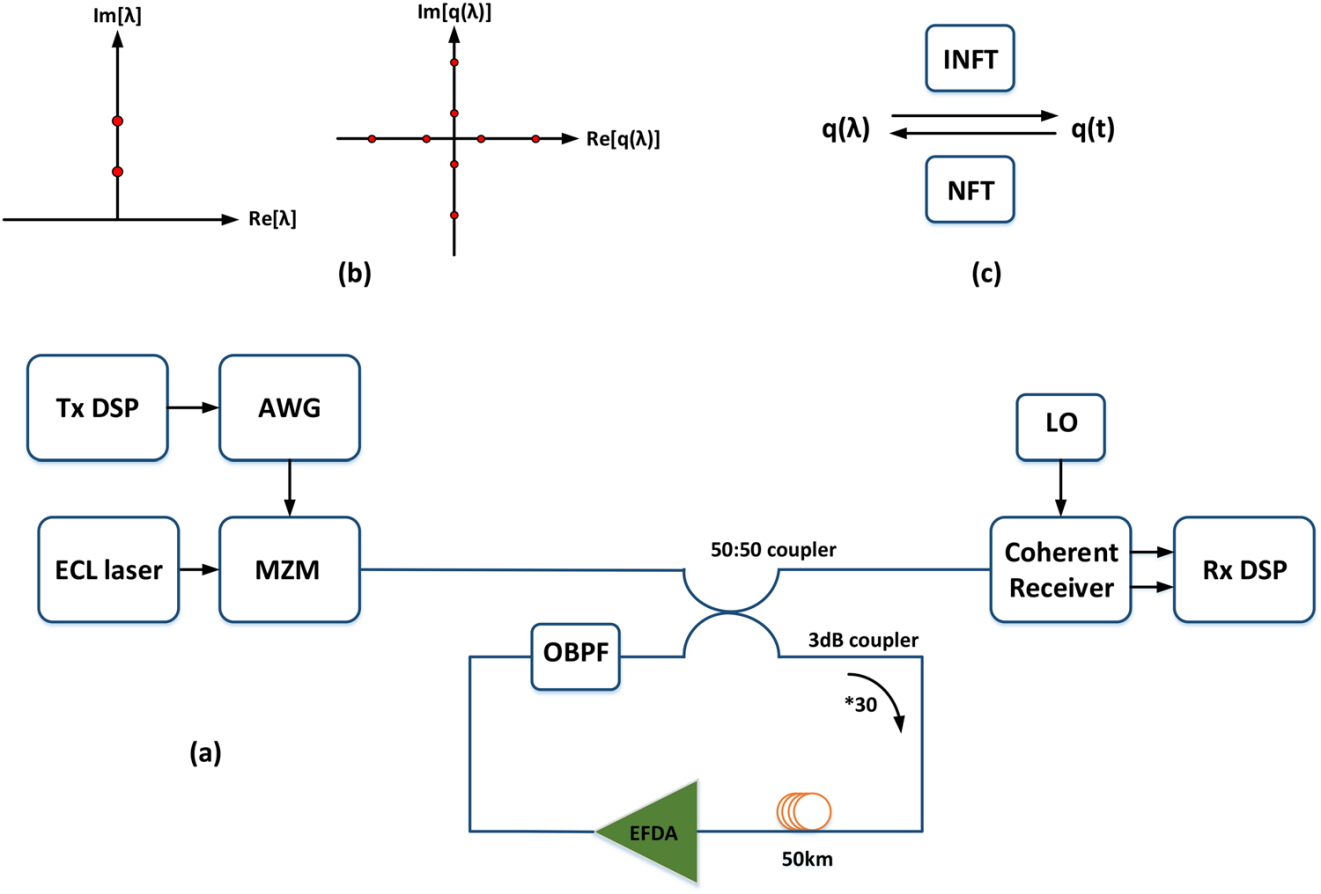
System setup which is used to verify spectral function modulation with two eigenvalues. (**a**) NFDM transmission system based on eigenvalue and spectral function modulation; (**b**) Designed eigenvalues and spectral functions in Tx DSP; (**c**) Correspondence of electrical pulse waveform in time domain and nonlinear frequency on the discrete spectrum.

The system setup for NFDM transmission is shown in Fig. 2. The transmitted signals are carefully designed in nonlinear frequency domain to help with easy decision at the receiver. By inverse nonlinear Fourier transformation (INFT) in Tx DSP module, these signals are converted to time domain and then generated by arbitrary waveform generator (AWG). Then the selected waveform is loaded onto the laser and launched into the fiber. For the integrability of the transmission equation, we used distributed Raman amplifier, while EDFA is used to compensate the residual loss of each span. After the coherent receiver, in Rx DSP module, NFT is used to convert the distorted signal to nonlinear frequency domain where nonlinearity removal is conducted. Then we can judge from both eigenvalues and spectral functions to decide transmitted signals.

**Figure 3:**
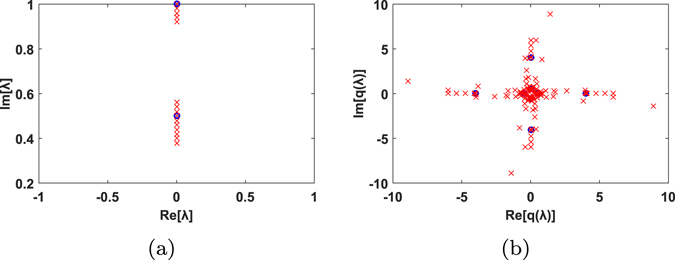
(**a**) Original eigenvalues at the transmitter (circle marks) and corresponding eigenvalues at the receiver(cross marks). All the waveforms here are generated with eigenvalues 1*i* and 0.5*i*. Eigenvalues suffer little distortion in transmission, which enables final signal recovery at the receiver. (**b**) Nonlinear spectral functions at the transmitter (circle marks) and at the receiver (cross marks). Different signals vary in their eigenvalues and spectral functions.

The recovered signals are analyzed in Fig. 3. Figure 3(a) shows the eigenvalues at the receiver (cross marks) compared to those at the transmitter (circle marks). It shows that eigenvalues suffer little distortion along the fiber. This property is deemed as the main principle of nonlinear transmission, enabling nonlinear compensation in DSP module. In Fig. 3(b), according to the received eigenvalues, we conduct nonlinear compensation and demonstrate received signals in the nonlinear spectrum as recovered spectral functions.

In signal decision module, when a signal is obtained after nonlinearity removal, we first check its eigenvalue so we can narrow the possibility down. Then we check the spectral functions to make the final decision. Using the above method, effective transmission can be realized.

## Discussion

### Strengths and weaknesses of combinations of eigenvalue and spectral function modulation

**Figure 4:**
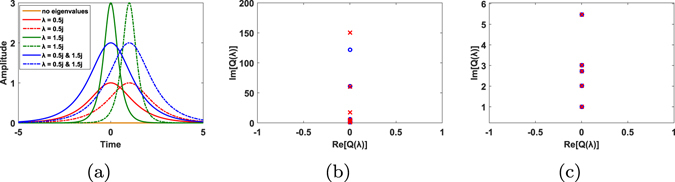
(**a**) Transmitted signals produced by INFT and time shift. (**b**) Nonlinear spectral functions at the transmitter (circle marks) and at the receiver (cross marks). Different signals vary in their spectral amplitudes. (**c**) Enlarged view of (**b**). Lower spectral amplitudes suffer less distortion in nonlinear frequency domain.

There are still two points to be highlighted. First, compared to eigenvalues, spectral functions are more susceptible to perturbations in transmission. From our simulation it shows that large spectral amplitudes can have more serious distortions than small ones, which restricts multi-level amplitude modulation. This is tested and demonstrated in Fig. 4. Two eigenvalues at 0.5*i* and 1.5*i* are used in eigenvalue multiplexing. Transmitting signals are shown in Fig. 4(a). The recovered spectrum is shown in Fig. 4(b). Signals with lower spectral amplitudes suffer less distortion along the fiber, while such distortion becomes more obvious with the increase in amplitudes. Figure 4(c) is the enlarged view of Fig. 4(b). It is also reported that phase modulation is sensitive to fluctuations^20^. So instead of using all the possible combinations for transmission, we choose 32 from all 80 signals so that the redundance provides fault tolerance in decision. Eigenvalue and spectrum provides double ways of accurate signal decision. Even if one of them suffers huge distortion, it is possible to distinguish signals from the other degree of freedom.

Second, this combination of modulation greatly improves spectral efficiency by enhancing degrees of multiplexing. In our case a total of 32 diverse signals are generated. However, advanced modulation schemes may be achieved using 16QAM or 32QAM, which means spectral efficiency can still be improved. It is also predictable that in further investigation we can combine this scheme with continuous spectrum modulation for higher capacity.

In conclusion, we propose a combination of eigenvalue and spectral function modulation, which provides a new method of nonlinear frequency division multiplexing. It exceeds the original single scheme of modulation by improving capacity as well as fault-tolerance.

## Method

Designing transmitting signals is the key part of the whole system. Since spectral data are more susceptible to noise and other losses in both electrical and optical domain, it is important that we choose constellations which can be easily detected at the receiver. We can restore the signal into time domain and launch it into the fiber. If designed properly, signals are more robust and suffer less distortion in transmission, so that they can endure longer distance. This provides us with new ideas about signal generation. Using NFT pair, we can conduct signal transformation with quick speed and convenience by computer.
